# Thoughts on Fatigue in Multiple Sclerosis Patients

**DOI:** 10.7759/cureus.42146

**Published:** 2023-07-19

**Authors:** Daphne Bakalidou, Vasileios Giannopapas, Sotirios Giannopoulos

**Affiliations:** 1 Laboratory of Neuromuscular and Cardiovascular Study of Motion (LANECASM) Physiotherapy Department, Faculty of Health and Care Sciences, University of West Attica, Athens, GRC; 2 Physical Therapy, University of West Attica, Athens, GRC; 3 2nd Neurological Department, Attikon University Hospital, Athens, GRC

**Keywords:** fatigue scales, fatigue, temperature, uhthoff's phenomenon, heat sensitivity, multiple sclerosis

## Abstract

Chronic fatigue is a common symptom in people with multiple sclerosis (PwMS) and presents as a reversible motor and cognitive impairment with reduced motivation and a desire to rest. The presentation of fatigue symptomatology in PwMS can be spontaneous or induced by mental or physical activity, temperature and humidity fluctuations, acute infections, and even food ingestion. Even though the exacerbation of fatigue symptomatology due to heat reaction is well established, the role of environmental temperature (ambient temperature and relative humidity) is not yet fully understood, and there is not enough systematic evidence regarding its effect. In this article, we present our opinion (based on the current literature and clinical experience) regarding the role of environmental temperature in the manifestation of fatigue symptomatology in PwMS.

## Editorial

Introduction

Multiple sclerosis (MS)-related chronic fatigue is a reversible motor and cognitive impairment with reduced motivation and desire to rest, either appearing spontaneously or brought on by mental or physical activity, humidity, acute infection, and food ingestion [1]. Fatigue in people with MS (PwMS) presents with lack of energy, inability to sustain or tolerate physical activity, feelings of malaise, and difficulties in concentration and task completion; however, each person may exhibit different combinations of fatigue-related symptoms [2]. Approximately 65% of PwMS experience fatigue symptomatology, which is directly correlated with independence levels, general quality of life, and disability levels. It is worth noting that 15-40% of PwMS who experience fatigue report it as the most disabling symptom compared to overall physical disability and pain [3,4]. Based on a recent study by Veldhuijzen van Zanten et al., fatigue is associated with fluctuations in depressive symptomatology, anxiety levels, perceived health status, and walking ability [5]. The effect of fatigue on the patient’s psychological well-being can be attributed to its interference with their responsibilities, work, and family life [5]. Regarding the assessment of fatigue in PwMS, due to its subjective nature and the lack of a strict clear medical definition, fatigue is usually measured by subjective scales [1]. Notably, the Modified Fatigue Impact Scale, Fatigue Severity Scale, Multidimensional Fatigue Inventory, and Fatigue Scale for Motor and Cognitive Functions [6-8] provide a subjective, self-reporting evaluation of the patient’s experienced fatigue symptomatology due to MS. The pathophysiology and underlying mechanisms that lead to the development of chronic fatigue in MS are still unclear, but the expressed symptomatology is similar to other auto-immune disorders. For example, patients with systemic lupus erythematosus (SLE) experience similar fatigue symptomatology due to different underlying disease mechanisms. In SLE, the development of fatigue is associated with obesity, poor sleep quality, depression, anxiety, cognitive dysfunction, vitamin D deficiency, and comorbid conditions [9]. In the case of MS, there are four major theories regarding the possible disease mechanisms leading to fatigue; however, none have been confirmed (Table 1) [10-12].

**Table 1:** Theories regarding fatigue in MS PwMS, people with multiple sclerosis; MS, multiple sclerosis

Theory	
Structural damage	White and/or grey matter damage that leads to regional or whole brain atrophy
Inflammatory process	Several antigens and auto-antigens, as well as the reactivity between microbial antigens and auto-antigens, have been examined, but none have been confirmed. Pharmacological agents such as interferon-a (which is rarely used in PwMS) can also induce the inflammatory process
Maladaptive network recruitment during task performance	Activation of atypical and non-adaptive regions as well as impairment of neuromodulatory projections originating from the brainstem
Metacognitive interpretations of brain states	Dysfunction in interoception, network-level function, or perceived effort of movement, which leads to a feeling of “helplessness”

The role of temperature in MS fatigue symptomatology

Heat reactions in PwMS, also known as Uhthoff’s phenomenon, have been recorded and studied since 1890 and occur in 60-80% of PwMS. Uthoff’s phenomenon is defined as “a temporary, short-lived (less than 24 hours), and stereotyped worsening of neurological function among multiple sclerosis patients in response to increases in core body temperature” [13]. Specifically, a 0.5°C increase in core body temperature can trigger temporary vision loss and worsening symptoms, which generally occur after exposure to warm environments and/or during exercise and can last until the core body temperature returns to baseline values [14,15].

The current theory behind the effect of high temperature in the exacerbation of the patient’s symptomatology is that demyelination reduces nerve conduction velocity, which in turn leads to a conduction block rendering the axon’s ability to transmit impulses of low frequency but not high-frequency trains. Environmental heat, humidity, and exercise seem to exacerbate heat sensitivity in PwMS, which can lead to premature fatigue [15,16].

A recent cross-sectional study between patients with relapsing-remitting MS, secondary progressive MS, and healthy controls in a climate-controlled laboratory revealed that relapsing-remitting MS had higher body temperature, which was associated with worse general fatigue levels but not cognitive fatigue levels [17]. Beyond the core body temperature, the role of environmental conditions (temperature and humidity) in the manifestation and the severity of fatigue in PwMS is still being investigated. A retrospective study in a large sample of German PwMS showed that fatigue levels were more affected by mood changes than environmental conditions [18]. In contrast, a longitudinal controlled study, which examined the variations of fatigue levels (using the Fatigue Severity Scale) during the year in correlation to temperature and humidity levels in a small sample of Greek PwMS, showed that higher temperatures and higher humidity levels (based on the discomfort index) were correlated with increased fatigue levels, with 22% of the fatigue variance being attributed to MS [19]. A key difference between these two studies is the evaluation time frame and the temperature variation between seasons. Bakalidou et al. evaluated each patient four times in the span of the year (accounting for temperature and humidity fluctuation between seasons) and recorded a minimum temperature of 10.9°C and a maximum temperature of 29.4°C [18]. On the other hand, Grothe et al.’s evaluation did not have a standardized evaluation time frame (each participant was evaluated randomly from one to five times in a year), and they recorded a minimum mean temperature of 2°C and a maximum temperature of 18.6°C [17]. A more recent study conducted in a small sample of PwMS with varying disability levels aiming to determine the optimal aerobic exercise parameters to minimize exercise-induced central and muscle fatigue concluded that cooling the exercise environment limits the negative effects of central fatigue [20].

Discussion

The effect of heat sensitivity in PwMS is a multidimensional issue with environmental factors such as ambient temperature, humidity, and geography, as well as factors associated with each PwMS (core body temperature baseline, disability levels, and cardiovascular capability) playing a major role in the manifestation and the severity of fatigue symptomatology. Although the role of ambient symptomatology remains ambivalent, controlling the patient’s environment (mainly the ambient temperature and relative humidity) and keeping the core body temperature near the baseline measurements can help PwMS mitigate the negative effects of heat. Regarding the management of fatigue symptomatology in PwMS, common pharmacological interventions include amantadine, modafinil (a wake-promoting agent that selectively works in the hypothalamic pathways), armodafinil, pemoline, and aspirin (reduction of inflammation and thermoregulation); however, a more recent systematic review of pharmacological interventions suggest the lack of strong evidence in the reduction of fatigue symptomatology (with the exception of modafinil) [21,22]. Additionally, fluoxetine is commonly prescribed for the management of MS fatigue-related symptomatology due to its neuroprotective action, even though its mechanism of action is not fully understood, and there is no strong evidence supporting its “anti-fatigue” action [23]. In contrast, educational and exercise interventions, energy conservation, and alternative forms of exercise such as yoga present supporting and strong evidence in reducing fatigue-related symptomatology [22]. New technological advances like the cooling suit can help PwMS not only avoid any type of symptom exacerbation due to high ambient and core body temperatures but also exercise and stay active [23].

In our opinion, there is a need for further exploration of the relationship between fatigue and environmental factors in PwMS. Ambient temperature fluctuations between seasons, humidity fluctuations between seasons, duration (days) of highest and lowest temperature, and characteristics of the specific MS population regarding work-related conditions and lifestyle trends are some of the factors that should be considered. Furthermore, secondary factors, such as mental status, quality of life, direct sun exposure, and sleep quality, in addition to the patient’s tendency to organize their routines in ways that can minimize fatigue due to climatic conditions have been speculated but not properly investigated [24,25]. Taking into consideration that in many places around the world, the median ambient temperature can surpass 35°C for more than two months per year, leading PwMS to be indoors and inactive. It is imperative to better understand the underlying mechanisms and relationships between heat and fatigue to find ways around it. The scientific investigation should not only embrace all aspects of the fatigue scales in use but also use objective measurements (core body temperature, VO2 max, etc.) to better record, evaluate, and analyze the manifestation, type, and severity of fatigue in PwMS.
